# Facilitators and barriers to implement nurse-led interventions in long-term dementia care: a qualitative interview study with Swiss nursing experts and managers

**DOI:** 10.1186/s12877-021-02120-1

**Published:** 2021-03-05

**Authors:** Julian Hirt, Melanie Karrer, Laura Adlbrecht, Susi Saxer, Adelheid Zeller

**Affiliations:** 1 Competence Center Dementia Care, Institute of Applied Nursing Sciences, Department of Health, Eastern Switzerland University of Applied Sciences (formerly FHS St.Gallen), Rosenbergstrasse 59, 9000 St.Gallen, Switzerland; 2 International Graduate Academy, Institute of Health and Nursing Science, Medical Faculty, Martin Luther University Halle-Wittenberg, Magdeburger Strasse 8, 06112 Halle (Saale), Germany

**Keywords:** Dementia, Implementation science, Evidence-based nursing, Barriers, Facilitators, Switzerland

## Abstract

**Background:**

To support the implementation of nurse-led interventions in long-term dementia care, in-depth knowledge of specific supporting factors and barriers is required. Conditions and structures of caring for people with dementia differ widely, depending on the country and the care context. Our study aimed to describe the experiences and opinions of nursing experts and managers with regard to facilitators and barriers to the implementation of nurse-led interventions in long-term dementia care.

**Methods:**

We conducted a qualitative descriptive study using individual interviews based on qualitative vignettes as a useful stimulus to generate narrations allowing to study peoples’ perceptions and beliefs. The study took place in nursing homes in the German-speaking part of Switzerland and in the Principality of Liechtenstein using purposive sampling. We intended to conduct the interviews face-to-face in a quiet room according to the participant’s choice. However, due to the lockdown of nursing homes during the COVID-19 pandemic in spring 2020, we performed interviews face-to-face and by video. We analysed data thematically following Braun and Clarke to achieve a detailed, nuanced description. To verify our interpretation and to ensure congruence with participants’ perspectives, we conducted member checks. The Standards for Reporting Qualitative Research (SRQR) served to structure our manuscript.

**Results:**

Six dyads of nursing home managers and nursing experts from six nursing homes took part in our study (*n* = 12). Our thematic analysis yielded seven themes reflecting facilitators and barriers to implementing nurse-led interventions in long-term dementia care: «A common attitude and cohesion within the organization», «Commitment on several levels», «A needs-oriented implementation», «The effect and the public perception of the intervention», «A structured and guided implementation process», «Supporting knowledge and competencies», as well as «Resources for implementing the intervention».

**Conclusions:**

To support the implementation of nurse-led interventions in long-term dementia care, active commitment-building seems essential. It is necessary that the value of the intervention is perceptible.Commitment-building is the precondition to reach the persons involved, such as nursing home managers, nursing staff, residents and relatives. Furthermore, nurses should precisely inform about the intervention. It is necessary that the value of the intervention is perceptible. In addition, nurses should adjust the interventions to the situational needs of people with dementia, thus. Therefore, it is important to support dementia-specific competencies in long-term care. Findings indicate that the barrier is determined by the intervention and its implementation – and not by the behaviour of the person with dementia.

**Supplementary Information:**

The online version contains supplementary material available at 10.1186/s12877-021-02120-1.

## Background

Dementia care is characterised by a research-to-practice gap reflecting a discrepancy between research-based knowledge and the application of this knowledge in practice [1–3]. In a survey of registered nurses in the field of elderly care, only one-fifth stated that they implement specific research findings in practice [4]. This is alarming since we know that not using evidence-based knowledge in clinical practice leads to lower quality of care for people with dementia [5, 6]. Therefore, implementing evidence-based interventions in clinical practice is a key aspect to reduce the research-to-practice gap [7]. The success of an intervention depends on various factors that can be described as facilitators and barriers.

Implementing interventions in dementia care is complex and challenging since they comprise multiple and multifaceted interacting components. Furthermore, different groups (people with dementia, health professionals, informal caregivers, care organisations) might receive and deliver interventions consisting of several components aimed at different outcomes [1, 8–10]. A current scoping review highlights supporting factors and barriers to the implementation of nurse-led interventions in dementia care. Thereby, nurse-led interventions were defined as interventions predominately performed by nurses [11]. Specific influencing factors for a successful implementation of nurse-led interventions in dementia care were (i) a person-centred organisational culture, (ii) a unified institutional culture and good communication among caregivers, (iii) collaboration between nurses and family members, (iv) factors directly associated with people with dementia (e.g. symptoms, behaviours, progressive loss of cognition, rapidly changing behaviours and situations), as well as (vi) financing and regulatory issues. These results provide a first picture of influencing factors particularly relevant for nurse-led interventions in dementia care. However, the scoping review did not yield sufficient details about these influencing factors. For example, details on the institutional culture’s attributes and forms of encouragement are lacking. There is no information about how to achieve and provide a person-centred organisational culture. Furthermore, it remains unclear how the influencing factors could be considered in the structure of the implementation process in long-term care and which aspects should be focused on. Detailed information about facilitators and barriers would be beneficial to support a sustainable implementation of nurse-led interventions in long-term dementia care [1, 11].

To support the implementation of nurse-led interventions in long-term dementia care, in-depth knowledge of specific supporting factors and barriers is required. Conditions and structures of care for people with dementia differ widely, depending on countries and care contexts [12]. It remains unknown whether known supporting factors and barriers can be adapted to the conditions of Swiss long-term care. Moreover, it is not known whether there are other and/or further influencing aspects regarding the implementation of nurse-led interventions in long-term dementia care. Detailed information about influencing factors for nurse-led interventions in dementia care and about structural properties of the implementation process are missing.

## Aim

Our study aimed to describe the experiences and opinions of nursing experts and managers on influencing factors concerning facilitators and barriers to the implementation of nurse-led interventions in long-term dementia care. The following research question guided our study: What are facilitators and barriers to the implementation of nurse-led interventions in long-term dementia care? The results may guide dementia care research supporting the implementation of nurse-led interventions in long-term care.

## Methods

### Study design

We chose a qualitative, descriptive design allowing the exploration and description of participants’ experiences and understanding of phenomena [13, 14]. Qualitative vignettes are known to be a useful stimulus to generate narration allowing to study peoples’ perceptions and beliefs [15]. Therefore, we conducted individual interviews based on qualitative vignettes. We analysed data thematically to achieve a detailed and nuanced description [16, 17]. To structure our manuscript, we used the Standards for Reporting Qualitative Research (SRQR) [18].

### Research team

Our research team consisted of senior researchers and PhD students in nursing and health sciences. All researchers directly involved in data collection and analysis are well-experienced in qualitative interviewing and/or thematic analysis. We are affiliated with a local Swiss university of applied sciences. Four of us live in Switzerland. All researchers have worked as registered nurses with people with dementia in Austria, Germany, and/or Switzerland.

### Setting and participant selection

The study took place in nursing homes in the German-speaking part of Switzerland and in the Principality of Liechtenstein. We used purposive sampling to identify dyads of nursing experts and nursing home managers from the same nursing home with in-depth knowledge about implementing interventions in dementia care and willing to critically reflect and share their experiences and opinions. Dyads were of interest since we wanted to gather perspectives on both the clinical and managerial part of nurse-led implementation. Eligible professionals (a) had to work as nursing experts (or in a position with a similar area of responsibility) or as nursing home managers (b) in a long-term care facility and (c) had to have expertise and experience in implementing nurse-led interventions. In Switzerland and the Principality of Liechtenstein, nursing experts and nursing home managers are responsible for implementing nurse-led interventions in long-term care institutions. They initiate projects, carry them out or they accompany the implementation of interventions during research projects in practice. In addition, they are responsible for deciding whether to continue with an intervention once it has been implemented.

Nursing homes were eligible if they had a specialised ward for people with dementia. To enhance variation in sampling, the nursing homes involved were run either publicly or privately. They were part of a national organisation, a local consortium or represented an individual institution. We recruited eligible persons by contacting the nursing home managers of corresponding nursing homes in the German-speaking part of Switzerland and in the Principality of Liechtenstein via email or by phone. If they (and the nursing expert of the institution) were interested in participating in the study, they received written information about the project. Participation in the study was voluntarily. To be included in the study, both nursing home managers and nursing experts of each interested institution had to sign a consent form. We knew some nursing home managers and nursing experts from previous projects. However, we did not have intensive contact with them before the study. We contacted seven nursing homes, six of which showed interest and agreed to participate in our study. One institution did not react to our request.

### Ethics

The ethics committee of Eastern Switzerland (Ethikkommission Ostschweiz, EKOS) approved the study (BASEC Nr. Req-2019-01288; EKOS 19/192). All participants signed the informed consent form.

### Data collection

We collected data by means of individual interviews using qualitative vignettes as a narration stimulus with follow-up questions. The vignettes and interview guidelines for nursing experts and nursing home managers are provided in the supplementary material. At the beginning of the interview, we introduced ourselves (name, professional background, research field and experience) and explained the study aim. Furthermore, we reminded the participants that there are no right or wrong answers and that we are interested in their personal opinions and experiences. We informed them that they can stop the interview and withdraw from the research at any time. After answering the participants’ questions, we presented the vignette. Vignettes are usually short stories of fictive persons representing example situations. Participants can relate and respond to them [15, 19, 20]. The vignettes in the current study consisted of the story of a fictional nursing expert or a nursing home manager, who wants to implement an intervention (Basal Stimulation) for people with dementia in the nursing home. We presented the vignettes in text form and requested the participants to read it at the beginning of the interview. The vignettes included a narrative prompt at the end. After reading the vignette, participants started to describe how they would proceed and what they would consider. Subsequently, we asked follow-up questions, thereby aiming to collect participants’ views on all relevant issues. The questions dealt with the following topics associated with dementia care: facilitators and barriers to implementation, facilitators and barriers to long-term implementation and sustainability of interventions, indicators for successful implementation and significant aspects of a successful implementation. LA, MK, SAX and AZ developed the vignettes and follow-up questions by drawing from previous research on this topic [11], thereby reflecting the local context. Two persons not involved in the project (a nurse expert and a researcher with expertise in vignette-based interviews), checked the vignettes and the follow-up questions for plausibility and meaningfulness. We adapted the vignettes according to their feedback. LA and MK (both PhD students) as well as SAX and AZ (both senior scientists) conducted the interviews between February and June 2020. We intended to perform the interviews face-to-face in a quiet room according to the participant’s choice. However, due to the lockdown of the nursing homes during the COVID-19 pandemic in spring 2020, we performed the interviews face-to-face or by video. However, the results showed that the mode of the interview did not influence its length, content and richness. The interviews lasted between 50 and 80 min, were audiotaped and afterwards transcribed. We took field notes to record participants’ non-audiotaped statements. A master student of nursing science and a staff member of our university transcribed the interviews. LA and MK checked and corrected the transcripts.

### Data analysis

The data analysis followed the six steps of thematic analysis according to Braun and Clarke [16, 17]. The method allows inductive identification, analysis and straight description of topics within data [21]. As a first step, we familiarised ourselves with the data by rereading the transcripts and noting first memos. Secondly, we coded interesting aspects dealing with our research aim in each interview. In the third step, we sorted the codes of the entire data set by looking for common aspects, patterns or connections and collected them under potential themes. We then (step 4) reviewed the themes according to their consistency with the coded text and the entire data set. Afterwards (step 5), we refined and named the themes. We described relationships and displayed them in a thematic map. In the sixth and last step, we wrote down the results. LA, MK, and JH performed the analysis, supported by critical discussions with SAX and AZ until we reached consensus. We used MAXQDA 2020 to conduct the analysis.

### Trustworthiness

To enhance authenticity and credibility, we used purposive sampling with the aim to recruit participants who can provide in-depth information relevant to the research topic. During the interviews, we paid close attention to participants feeling safe and free to express their points of view. To ensure that participants’ statements are accurately captured, we mutually critically reviewed data-driven coding in multiple alignment and harmonisation sessions. Two researchers and a professional translator double-checked the translation of quotes embedded in our article. All researchers have worked as registered nurses with people with dementia. We therefore critically reflected our experiences and background before and during data collection and analysis.

To verify our interpretation and to ensure congruence with participants’ perspectives, we conducted a member check [22, 23]. Following our invitation, two nursing experts and one nursing home manager from two nursing homes consented to participate. AZ sent the results of our study to the participants with the request to comment on (dis)agreement, comprehensibility, and incomprehensibility [24]. After approximately 2 weeks, AZ collected the feedback in a scheduled personal phone call with each participant of the member check. AZ and JH discussed the feedback and inserted minor changes corresponding to participants’ feedback.

## Results

### Participants’ characteristics

Six dyads of nursing home managers and nursing experts from six nursing homes took part in our study (*n* = 12). Sociodemographic and professional characteristics per participant are shown in Table 1. Table 2 illustrates characteristics of included nursing homes.

**Table 1:** Participants’ sociodemographic and professional characteristics

Nursing home (#)	Function in the nursing home	Age (years)	Work experience in nursing (years)	Work experience in the current position (years)	Work experience in the current nursing home (years)	Qualification in addition to basic nursing training: further training (position-specific or dementia-specific)
1	Nursing home manager	55	29	8	10	Nurse manager education, Higher Education for Health Care Professions Grade I, Trainer with a Swiss Federal Vocational Diploma
1	Nursing expert	41	22	6	10	Graduated in Nursing Science, university course in «Organisational Ethics», Master of Science in «Nursing Management»
2	Nursing expert	53	33	15	10	Master of Advanced Studies in «Gerontological Nursing»
2	Nursing home manager	49	28	11	11	Certificate in «Management in Health Care Systems»
3	Nursing expert	58	36	2.5	2.5	Certificate of Advanced Studies in «Dementia and Way of Life»
3	Nursing home manager	45	25	10	0.3	Certificate of Advanced Studies (3 programs)
4	Nursing expert	59	18	11	7	Course in Validation
4	Nursing home manager	40	19	5	4	“Postgraduate course «Team Management and Head of Department», Certificate of Advanced Studies in «Gerontological Care», Pain Management”
5	Nursing expert	43	20	12	7	Master of Science in Nursing, Trainer with a Swiss Federal Vocational Diploma, Validation Basis, Grade I and II, Aggression Management
5	Nursing home manager	55	38	29	1	Management training for Nurse Managers, Leadership Specialist with a Swiss Federal Vocational Diploma; Quality Management, various professional trainings in the field of dementia
6	Nursing home manager	50	30	23	20	“Master of Advanced Studies in «Management of Health Care Institutions», Nurse Expert Higher Education for Health Care Professions Grade II, Validation “
6	Nursing expert	60	40	20	20	Master of Advanced Studies in «Mental Health», Trainer with a Swiss Federal Vocational Diploma, Certificate of Advanced Studies in «Gerontological Psychiatry»

**Table 2:** Characteristics of included nursing homes

Nursing home (#)	Nursing home residents (n)	Residents living in a specialised ward for people with dementia (n)	Specialised wards for people with dementia (n)
1	245	41	3
2	223	18	2
3	147	44	3
4	72	48	2
5	131	24	1
6	300	90	4

### Themes

Our thematic analysis yielded seven themes reflecting influencing factors concerning facilitators and barriers to implementing nurse-led interventions in long-term dementia care: «A common attitude and cohesion within the organization», «Commitment on several levels», «A needs-oriented implementation», «The effect and the public perception of the intervention», «A structured and guided implementation process», «Supporting knowledge and competencies», as well as «Resources for implementing the intervention» (Table 3).

**Table 3:** Themes reflecting influencing factors to implementing nurse-led interventions in long-term dementia care

A common attitude and cohesion within the organization	
Commitment on several levels	
A needs-oriented implementation	
The effect and the public perception of the intervention	
A structured and guided implementation process	
Supporting knowledge and competencies	
Resources for implementing the intervention	

#### A common attitude and cohesion within the organization

Aspiring a collective development in an organization is favorable for implementing an intervention. Collective development comprises a vision and values shared by all members. Only under this condition, all persons can contribute with their individual work to the total output.

*«We need common values, visions and strategies. It should be clear that we all work for a common cause and everybody has a role»* (nursing home 6, nurse manager, section 53)

To ensure a collective development, it is necessary to support cohesion and a common attitude. All persons involved should regularly enter into a dialogue with one another and cultivate positive relationships. Therefore, it is required to openly address conflicts and to jointly elaborate projects. This also contributes to team development and facilitates a common attitude in the team as an important prerequisite for successful implementation.

To create an organizational culture characterized by cohesion among all persons involved, it is necessary that relatives are considered as an integral part of dementia care. Under this condition, relatives may feel themselves involved. Therefore, all persons affected by the intervention can move in the same direction. Conversely, discrepancies in an organization may be a barrier to successful implementation.

The attitude towards the implementation on the part of the organization and of the persons involved is essential for successful implementation. Therefore, it is important that the intervention is in accordance with the mission and the values of an organization. This implies to determine one or a few high-priority issues. As a result, this focused approach may contribute to organizational coherence. It may facilitate a common attitude towards a specific issue.

*«It is better to focus on one issue and to say: We are going to implement this. We are ready to provide resources. We are going to pursue this in the long run. We really want to live that in everyday life. And we are going to evaluate it. Thinking in long terms … I believe that this is more helpful because people learn something. They have a sense of achievement and they enjoy working together on an issue that has finally become familiar to them»* (nursing home 2, nursing management, section 12)

In case of too many issues, priorities are not clear, an overview is missing, and issues lose attention at an early stage. This impairs a sustainable implementation.

#### Commitment on several levels

To successfully implement interventions, the commitment of all persons involved is required. Persons in leadership positions, nurses as well as residents and their relatives should approve the intervention. To ensure this, active commitment-building is necessary. Participants particularly underlined the necessity of involving nurses working in direct contact with residents in order to motivate them for the implementation of the intervention:

*«I believe that the success stands or falls with the enthusiasm of the nurses. It is necessary that they really understand what it is all about. And that they are motivated to put it into practice. That’s very demanding»* (nursing home 4, nursing expert, section 29)

Accordingly, it is essential that the nurses involved understand the intervention. In case of doubts, it is required to address their reservations and to actively strengthen the motivation of every nurse, for example by offering the possibility of personal development or by taking over responsibility within the project. If nurses are not involved, this can result in oppositional behavior and in doubts preventing successful implementation. Conversely, nurses with an open-minded attitude towards innovation approve the project with enthusiasm and contribute to successful implementation.

*«Persons with dementia are quite sensitive on the emotional level. They immediately feel whether the nurse is fully committed or not. The nurse should be convinced that she is doing the right thing. Then she helps the person with dementia to feel safe»* (nursing home 4, nursing expert, section 55)

Apart from nurses working in direct contact with residents, support from persons in leadership positions is crucial as well. This concerns members of the executive management, nursing managers and ward managers. Their approval is a major precondition for starting the project, for receiving necessary resources, for ensuring the liability and seriousness of the project, as well as for sustainable implementation.Furthermore, not only professionals but also persons with dementia and their relatives should approve the intervention. Particularly, relatives might have a supportive role since it is often not possible to inform the person with dementia about the intervention in a way she/he fully understands. In addition, people with dementia probably might not be able to clearly express consent or refutation. In this case, relatives can contribute to assess how far an intervention conforms to the will of the person with dementia.

However, if the person with dementia and her/his relatives are generally sceptical about the intervention, this may be a barrier to implementation. This could happen if relatives consider the intervention inappropriate or not beneficial for the resident. A high need for rigid structures on the part of persons with dementia or relatives could also be an obstacle. Conversely, curiosity may facilitate the implementation.

#### A needs-oriented implementation

The intervention and its parts should be described in a transparent, understandable and comprehensible way. From the participants’ point of view, this is favorable for the implementation. A well-structured instruction can be helpful. Additionally, flexibility and situational adjustment are important as well. Therefore, it is required to tailor the intervention to the physical, emotional, cognitive and social needs of the person with dementia. There should be sufficient freedom for targeted adaptations.

*«The status of a person with dementia is very volatile. Sometimes mental stability changes within seconds. Or they show different moods on the same day. That’s extremely challenging. It‛s difficult to choose the right time. You can‛t say: Today I‛m going to do this and that. You always have to adapt it to the current situation. You can‛t strictly follow your plan»* (nursing home 4, nursing expert, section 93)

There are different options for adjusting an intervention to the current situation of a person with dementia. On the one hand, temporal modifications (i.e. duration of the intervention) are possible. On the other hand, individual adaptations (specifically tailored to the person with dementia) may be helpful.

*«It is essential that the person is ready as well. Especially in the context of Basal Stimulation. And particularly when you work with aroma essences. Naturally, this is very individual. You have to adapt it to the situation. That’s crucial»* (nursing home 4, nursing expert, section 17)

The implementation threatens to fail if professionals do not recognize the needs of the person with dementia or if they ignore these needs. It is essential to consider the stage of disease. Dementia is associated with progressing and/or fluctuating physical, emotional and cognitive burden of disease, depending on the stage.

*«[Adaptations to the situation ]are necessary for all people. But for persons with dementia adaptations are particularly important. Otherwise, they probably respond very rejecting»* (nursing home 5, nursing management, section 55)

Therefore, situational peculiarities are highly important. To perform an intervention according to the needs of the person with dementia, health professionals have to evaluate the current situation and to initiate necessary adjustments. This requires the competence to assess current requirements of persons with dementia. Furthermore, there is also a need for readiness to plan nurse-led intervention autonomously, to implement them in a reflective way and to adapt them according to situational demands.

#### The effect and the public perception of the intervention

Whether an intervention has an effect and to what extent this effect is manifest, is important with regard to implementation. The interviews revealed that scientific evidence, positive experiences in the external realm and direct visibility of the effect in the field of practice may be supportive. According to the participants, the verified effect of an intervention in an evaluation study (external evidence) is important with regard to credibility. It may contribute to the final decision in favor of an implementation.

Additionally, it is supportive if an intervention has already been successfully implemented in another institution. Under this condition, it is possible to rely on previous experiences. Evidence-basing and positive reporting on the part of other institutions are the basis for an argumentation in favor of implementing an intervention. Previous experience may also have a motivating effect on all persons involved.

Internal evidence on the effect of an intervention has high significance with regard to implementation. In this way, the effect of the intervention is directly visible for persons involved. Positive experiences with the intervention may contribute to promote the implementation. If the intervention turns out to be ineffective, this can be frustrating for the persons involved and may prevent the implementation. Ideally, the intervention should evoke a positive response in persons with dementia. This response should be visible for nurses and relatives or there should be a possibility to show them this positive response.

*«It is important to experience the effect and to see it. I somehow experience these moments with the resident. Or the resident flourishes. She feels better or sleeps better. Then it‛s necessary to communicate this. And then I believe that it‛s easy to get the team on board very soon»* (nursing home 3, nursing manager, section 73)

Particularly with regard to complex interventions in the field of dementia care, an effect is not immediately visible. This may be challenging for the persons involved. High frustration tolerance is required.

*«If the issue doesn’t work, try it again! Perhaps for the eleventh time … You realize this with issues like validation. Many attend the course for three or five days. They learn a technique or the technique as such is already difficult. And then they are extremely frustrated because they can’t connect it with everyday practice, with their own evaluation of the stage. And in the end, they can’t understand it, they have questions»* (nursing home 1, nursing expert, section 59).

Furthermore, it is supportive if the intervention has not only a positive effect on persons with dementia but also on nurses and their work. The intervention may facilitate nursing procedures due to reduced resistance on the part of residents. It may also improve communication with persons with dementia.

To demonstrate the effectiveness, it is important to perform an evaluation. However, it can be challenging to determine the effectiveness of an intervention in persons with dementia. For this purpose, it is necessary to use subjective criteria. Due to dementia-related communicative or emotional barriers, these criteria may be difficult to assess. Therefore, it is essential to thoroughly observe the residents’ response to the intervention. Probably, different nurses estimate the effectiveness in various ways. For this reason, it is significant to prepare nurses for the evaluation.

If the effectiveness of the intervention excites the interest of external parties, this is supportive for the implementation. Ideally, an effective intervention also attracts the attention of potential residents and their relatives. In doing so, the institution could differentiate itself and earn an excellent reputation. According to the participants, such a positive external effect may also be attractive for nurses.

#### A structured and guided implementation process

According to the participants, it is important to begin the implementation process with detailed forward planning, for example with regard to project budgeting, clarification of already existing knowledge and scheduling of training sessions. The project team determines the aims of the intervention and defines how to evaluate the actual achievement of these aims. Prior to the start, project initiators intensively immerse themselves into project-related issues, thereby selectively involving other persons concerned.

*«There is a high need for communication in our teams. We have to announce that soon there will be something new and that we want to achieve a certain aim. We probably have to define the aims with the team leader. It’s important to decide what we want to achieve in which period of time. And we have to arrange certain things. Arrangements are necessary to proceed in a structured way»* (nursing home 2, nursing expert, section 30).

If a structured approach and the involvement of others are missing, this poses a barrier to implementation. A lack of forward planning may lead to deception and frustration. Therefore, starting with a pilot project seems to make sense, particularly in the case of more comprehensive projects. A pilot project allows to timely recognize difficulties concerning implementation. Participants mentioned that liability is necessary as soon as the project is implemented on a larger scale. Liability becomes evident, for example, in a project agreement, in anchoring the intervention into the organizational structure and in the evaluation of project aims. In case of success, it is necessary to celebrate and to communicate what the team has achieved.

If control and reflection are lacking, the project is in danger to lose visibility in everyday life. To prevent this, it is necessary to define persons responsible for the project. On the one hand, a functioning project group with an experienced leader is necessary. On the other hand, clinical multiplicators with expert knowledge and motivation are required to represent the project topic in the field of practice and to accompany the implementation. According to the participants, it is an inhibiting factor if the implementation is based only on individual persons.

Furthermore, persons responsible should openly communicate about the project from the beginning. They should accompany all persons involved during the implementation. To ensure that nurses and relatives can approve the project, it is necessary that they are well-informed about its content and aims. During the implementation process, nurses particularly need practice guidance offering the possibility of training and reflection. According to the participants, it is illusionary to assume that nurses will implement the concept by themselves after training. They emphasize that guided learning in practice is as important as training. This is particularly true for implementing complex interventions aiming to ensure a high level of liability.

*«At the beginning, you have to practice again and again. Until it really works. And someday it is self-evident, without saying. This is the case in two of our units. There’s no need to discuss anymore. Something that was very hard at the beginning is now just normal. I’s routine»* (nursing home 4; director, section 63)

#### Supporting knowledge and competencies

Already existing knowledge and competencies of nurses are necessary to implement an intervention. Additionally, time resources are required to exchange and to maintain knowledge related to the intervention. If nurses already have a highly developed professional standard in dementia care and they work according to their competencies, this is already a solid foundation for implementing dementia care interventions.

*«It’s necessary that nurses have developed the ability of crosslinked thinking … In this respect, our nurses are worth their weight in gold. In two 'segregated living' units, we have nurses with a long history of continuing education. For them, it is possible to link the new issue with the disease and its different types. Due to previous knowledge and basic knowledge, many things are much easier»* (nursing home 4, director, section 30)

According to the participants, competency also comprises an open-minded attitude on the part of nurses. Due to this fundamental attitude, the implementation of certain interventions proves to be much easier. As an example, the participants mentioned Basal Stimulation requiring a high level of motivation to concentrate on a person, as well as empathy and low fear of contact.

Already existing experience and concrete knowledge concerning implementation-associated issues are also favorable. From the participants’ point of view, it is essential to maintain and to extend what nurses already know. Therefore, the intervention should be regular topic. Organizations should ensure refresher courses. Moreover, exchange among nurses is favorable as well.

*«These issues should not lose our attention. There is a regular exchange in our standard groups. At least six times a year, regularly, until things tend to turn into a routine»* (nursing home 4, director, section 61)

To maintain knowledge and competencies in an organization, team stability and a low turnover rate are required. In case of high fluctuation, there is a risk of losing knowledge. In this case, the issue is not further developed any more. As a result, rebuilding knowledge is very time-consuming. Team stability and low turnover are a precondition for preserving knowledge. This also enhances the quality of relationships among nurses resulting in intensified, more open-minded exchange. Nurses giving each other feedback and exchanging their knowledge may have a positive impact on the intervention.

*«This works wonderfully on one of my units. Because they also give each other feedback. Positive and constructive feedback. Naturally, this is only possible when the team is holding the strings together»* (nursing home 1, nursing manager, section 34)

#### Resources for implementing the intervention

According to the participants, sufficient resources are favorable for implementing an intervention. This refers to sufficient financial, personal and temporal resources to ensure necessary attention for the implementation.

*«If you want to implement such concepts, staffing level is a big issue. The team needs certain resources. Whenever you implement something, you should dedicate enough time and attention to this project»* (nursing home 5, nursing manager, section 18).

Optimized staffing and sufficient temporal resources are needed. However, it becomes obvious that nursing homes fundamentally affected by staff shortage cannot operate without additional resources. Otherwise, there is not sufficient time for nurses to implement the intervention.

*«No matter how excellent the project is – if I have not enough staff or staff is always reduced, it‛s not effective. It‛s an additional strain on personnel. As much as they try to, they cannot implement it. […] And that’s the key problem […] Staff is necessary. Ideally, I would like to have more staff also in the field of long-term care. But in this area, the need is not yet recognized as urgent»* (nursing home 4, director, section 47)

From the participants’ point of view, costs for infrastructure or for training pose a major challenge for implementing interventions. Not every organization can fulfill infrastructural demands associated with a project. This results in additional demands and costs. The participants mentioned technical needs, a lack of room capacities as well as access to nursing databases. Satisfying technical equipment is not always ensured in nursing homes. Room capacities often are insufficient for training or information events. Particularly, a separate room for implementing an intervention is necessary. To change work processes associated with the implementation, training is necessary. This relates to nurses, teams or individual persons. They all need training in order to prepare the intervention and to perform it sucessfully.

## Discussion

Successful implementation of nurse-led interventions in dementia care is highly challenging due to the complex nature of interventions [10]. To design implementation strategies in an optimal way, it is important to know facilitators and barriers. From the perspective of nursing experts and managers, it is necessary to consider (i) whether there is a common attitude and cohesion within the organization, (ii) whether commitment is present on the institutional and staff level, (iii) and whether the implementation follows principles based on the needs of people with dementia. Furthermore, (iv) the effects of the intervention and the degree of its public perception is notable, as well as (v) a structured and guided implementation process, (vi) knowledge and competencies of staff, and (vii) sufficiently available financial, personal and temporal resources supporting the implementation process.

Our results confirm and expand the evidence concerning facilitating and hindering aspects of implementing nurse-led interventions in dementia care. A recent scoping review reported that organisational culture is essential for implementing interventions in dementia care as described in over 50% of the included studies. Particularly, a person-centred culture of care and flat hierarchies were identified as facilitating factors for the implementation of interventions [11]. A recent systematic review identified openness to change from the part of nursing home staff as an indicator for organisational culture with regard to successful implementation of interventions for people with dementia [9]. Our interviews with Swiss long-term dementia care nursing experts and nursing home managers provide further information about this aspect. The results highlight that the support of cohesion and a common organisational attitude are necessary to ensure a collective development. Therefore, all persons involved should regularly enter into a dialogue with one another and should cultivate positive relationships. Our findings further suggest that a shared vision and common values of all members depend on openly addressing conflicts and jointly elaborating projects. This might contribute to team development and to facilitate a common organizational attitude as important prerequisites for successful implementation.

According to our results, the commitment of all persons involved is required to successfully implement nurse-led interventions in long-term dementia care. Of crucial importance is support from persons in leadership positions, for example members of the executive management, nursing managers and ward managers. This was also highlighted in findings from the process evaluation of a randomised controlled trial on dementia care mapping [25]. Strong leadership might be a major precondition for starting the project, for receiving necessary resources, for ensuring the liability and seriousness of the project, as well as for sustainable implementation [9]. Furthermore, it seems important that nursing homes prioritize their commitment to implementing nurse-led interventions. Therefore, a clear strategy might be helpful in order to avoid multiple projects running simultaneously. For this purpose, a straight focus on the current intervention and its implementation is necessary [26].

Being convinced about the intervention’s value on the part of the staff was emphasised as a precondition for a successful implementation [27]. Our results confirm this aspect and expand it. They show the importance of proven effectiveness of the intervention in similar and comparable situations. This aspect proved to be important especially for nurses. External evidence concerning the effectiveness or the successful implementation of the intervention in another nursing home are of central importance. In addition, it seems to be meaningful for nurses that the effect of the intervention can be directly observed and its benefit can be directly derived. However, data concerning these aspects are not always at the nurses’ disposal. To assess the impact of a specific intervention, training measures could be a possible support. This should be coupled with guidance on ways of obtaining information about the benefits of an intervention.

As indicated by a recent scoping review [11], fluctuating needs and behaviours of people with dementia are barriers to a successful implementation of nurse-led interventions in dementia care. In our qualitative study, these two aspects also emerged as a challenge and requirement with regard to implementation. The intervention must be adaptable to the individual needs of people with dementia [27]. At the same time, nurses should be able to adjust an intervention after they have implemented it. With regard to flexibility and necessary situational adjustments, there are high demands on (dementia-specific) nursing competencies. Thus, our findings indicate that the barrier is determined by the intervention and its implementation – and not by the behaviour of the person with dementia. Consequently, a high level of training for nurses could be a key aspect of implementation [27, 28]. To ensure well-trained staff, permanently accessible in-house training structures are needed. This is associated with financial, time and human resources [9]. It is doubtful whether the level of nursing competency in long-term care in Switzerland is sufficient [29]. Since the composition of qualification levels for nursing staff is regulated on a cantonal basis, there are differences within Switzerland. In the canton in which the majority of the interviews took place, qualifications required for nurses working at the management level are defined by the operator of a nursing home, not by law [30]. The nurse-to-resident ratio is regulated by law conforming with the residents’ care dependency level. The proportion of nursing staff trained at tertiary level (corresponding to academic education on a bachelor level) and at upper secondary level (corresponding to nursing diploma) is also defined. However, the proportion of these educational levels is at least 40% and it is challenging for nursing homes to find well-educated nursing staff [31]. Therefore, it is unclear to what extent nursing educational levels meet the requirements to adapt nurse-led interventions to the needs of persons with dementia. Since some persons with dementia have extensive care needs, it can be highly challenging for a nursing home to meet demands on implementation [32, 33].

A scoping review identifies the engagement of families as an important factor contributing to the successful implementation of interventions in dementia care [11]. According to our interviews, the relatives’ commitment to the intervention is crucial. Furthermore, our findings offer in-depth information about this aspect. They emphasize the importance of active commitment-building on the part of the healthcare professionals. Thus, the collaboration of key persons involved in the implementation process (e.g. nursing experts, nursing home managers) with relatives of people with dementia is an important aspect. According to our results, it is necessary that relatives feel involved and are well-informed about the intervention and the way it is implemented. As a result, relatives may develop a positive attitude towards a specific intervention. In this case they might have a supportive role in informing the person with dementia about the intervention in a comprehensive way. Furthermore, relatives can contribute to assess how far an intervention conforms to the will of the person with dementia. A recent qualitative study on the experiences of families collaborating with staff [34] also highlighted the important role of relatives. Good communication between staff and family proved to be a key factor in order to improve collaboration and to create partnerships with families. Informal contact between staff and family members, informing family members and inviting them to participate in organised communal activities were characteristic for good communication. When family members are demanding and probably do not consider that nurses are obliged to care for a group of people and not only for one single person with dementia, nursing staff may experience a difficult relationship with family members [35–37]. This might have a negative influence on building and keeping partnerships between family members and nursing staff [38, 39]. However, more primary research focusing the effectiveness of interventions aimed at creating and strengthening family-staff partnerships is needed. As indicated by a recent systematic review on interventions to foster family inclusion in dementia care [40], only few studies address this topic. Furthermore, studies should carefully consider the perspective of both nursing staff and family members on a given intervention, e.g. by conducting an exhaustive process evaluation of the intervention [41].

It is a strength of our study that we recruited participants with expertise and experience in implementing nurse-led interventions. They are responsible for the implementation process and for the decision whether to continue an intervention after its implementation. The recruitment of dyads (nursing experts/nursing home managers) from the same nursing home allowed a comprehensive perspective based on in-depth knowledge concerning the clinical and managerial part of implementing nurse-led interventions in dementia care. Since we used qualitative vignettes, we were able to stimulate narration. Follow-up questions allowed to ask for additional information based on participants’ in-depth knowledge. Furthermore, several experienced coders performed thematic analysis following Braun and Clarke [16, 17]. We used member checking to verify our interpretation and to ensure congruence with participants’ perspectives [23].

Some methodological limitations are obvious and require attention. First, due to project resources, our sample size was limited to a region in Eastern Switzerland and the Principality of Liechtenstein. Second, we used purposive sampling and we are aware that recruitment of participants is therefore selective. However, we searched for variation in sampling and involved nursing experts and managers from nursing homes that were publicly or privately run, part of a national organisation, a local consortium or represent an individual institution. Third, due to project time resources and COVID-19 pandemic delays, our sample size was limited to twelve participants. These limitations need to be reflected with regard to the transferability and generalizability of our results.

## Conclusions

Our results provide support for researchers and other key persons involved in implementing nurse-led interventions in long-term dementia care by offering in-depth information about facilitators and barriers to the implementation of nurse-led interventions in dementia care. Active commitment-building seems essential to reach the persons involved, such as nursing home managers, nursing staff and residents as well as their relatives. Responsible persons can actively influence the implementation process by supporting cohesion and a common organisational attitude based on shared values. Furthermore, nurses should precisely inform about the intervention. The value of the intervention should be perceptible. Thereby, establishing supporting relationships with relatives proves to be an important factor. To ensure successful and sustainable interventions, adjustments to the situational needs of people with dementia are essential. For this purpose, a high level of flexibility, knowledge and competencies on the part of the staff is required. Thus, besides adequate financial and time resources, it is important to support dementia-specific competencies in long-term care.

## Supplementary Information

**Additional file 1.**

## Data Availability

The anonymised datasets are available from the corresponding author on reasonable request.
