# Supplementary material for: Facilitators and barriers to implement nurse-led interventions in long-term dementia care: a qualitative interview study with Swiss nursing experts and managers
Source: BMC Geriatr. 2021 Mar 5;21:159. doi: 10.1186/s12877-021-02120-1 (PMC7932832; doi:10.1186/s12877-021-02120-1)
Supplement: Supplementary file 1 — Additional file 1. [file 12877_2021_2120_MOESM1_ESM.pdf]

SUPPLEMENTARY MATERIAL

Facilitators and barriers to implement nurse-led interventions in long-term dementia care: A qualitative interview study with Swiss nursing experts and managers

Julian Hirt [1,2*], Melanie Karrer [1*], Laura Adlbrecht [1], Susi Saxer [1], Adelheid Zeller [1]

[1] Competence Center Dementia Care, Institute of Applied Nursing Sciences, Department of Health, Eastern Switzerland University of Applied Sciences (formerly FHS St.Gallen), Rosenbergstrasse 59, 9000 St.Gallen, Switzerland

[2] International Graduate Academy, Institute for Health and Nursing Science, Medical Faculty, Martin Luther University Halle-Wittenberg, Magdeburger Strasse 8, 06112 Halle (Saale), Germany

[*] Authors contributed equally.

Corresponding Author

Adelheid Zeller, Competence Center Dementia Care, Institute of Applied Nursing Sciences, Department of Health, Eastern Switzerland University of Applied Sciences (formerly FHS St.Gallen), Rosenbergstrasse 59, 9000 St.Gallen, Switzerland, heidi.zeller@ost.ch

<u>Procedure of the interview (case vignette and interview guideline): nursing managers</u>

Introduction

- Introducing oneself to the participant
- Informing about the aim: We would like to explore how interventions in the field of dementia care are implemented and what kind of challenges are associated with this in practice.
  By the term «intervention» we mean a nursing activity or a concept that has been implemented in practice with the aim to improve the quality of care for people with dementia.
- To figure this out, we would like to interview nursing managers and persons who implement interventions in nursing homes.
- Informing about the procedure: First, I will ask you to read a story, to immerse yourself into this story and to tell me what you think about it. After that, I would like to ask some more detailed questions. You are welcome to bring in your point of view as nursing managers. I will record this interview in order to transcribe it afterwards.
- The interview will take approximately one and a half hours.
- Do you have any questions before we start? Haben Sie vorab noch eine Frage?
- *(Procedure: We handed out the vignette and asked the participants to read it. Then we presented a graph about Basal Stimulation and briefly explained each of the points)*

Case vignette:

Miss Huber is nursing manager in a nursing home in Eastern Switzerland. At a congress of the Swiss Professional Associations of Nurses, she listened to a lecture about Basal Stimulation. Since then, she is convinced that by means of this concept persons with dementia could experience better care. So far, in her institution no actions have been taken in this direction. This is a cause for concern and Miss Schmid is alarmed. She cannot get out of her mind the possibilities associated with this concept for persons with dementia in her institution. She expects positive consequences, for example improved body awareness and reduced agitation. Probably her colleagues could also benefit from this concept, particularly with regard to interventions in the context of physical care for persons with aggressive or agitated behavior. The whole institution might benefit since Basal Stimulation could also have an impact on the attitude of all professionals. She deliberates whether it would be possible to integrate this concept on the specialized ward for persons with dementia.
What should she do? How could she realize this? On the next day during a coffee break, she meets the nursing expert who is responsible for implementing projects. She enthusiastically tells her about the lecture and her ideas …

Procedure: First, we ask the guiding questions and wait for the answers. Afterwards, we ask the concrete questions, as far as they are still necessary.

Guiding questions	Concrete questions
What could be the next steps in order to launch such a project?	What are the focal points of your deliberations?<br>In what way you would be involved?<br>Which other persons on which levels would be involved?<br>Which steps seem important to you?<br>Which resources would be required?<br>What should you have to take into consideration (principally)?<br>What requires particular consideration?<br>Which basic conditions would be helpful?<br>How did the institution proceed in similar projects?
Which obstacles or barriers do you perceive?	Which obstacles do you perceive<br>… on the organizational level?<br>… on the level of nurses/health professionals?<br>… on the level of persons with dementia?<br>… on the level of relatives/on the family level?
What could facilitate the implementation of the project from your point of view?	What facilitates the implementation of the project<br>… on the organizational level?<br>… on the level of nurses/health professionals?<br>… on the level of persons with dementia?<br>… on the level of relatives/on the family level?
What is particularly important to successfully implement the project from your point of view?	Which aspects are significant from your point of view<br>… on the organizational level?<br>… on the level of nurses/health professionals?<br>… on the level of persons with dementia?<br>… on the level of relatives/on the family level?
How do you recognize the successful implementation of the project?	How do you recognize the successful implementation of the project<br>… on the organizational level?<br>… on the level of nurses/health professionals?<br>… on the level of persons with dementia?<br>… on the level of relatives/on the family level?
What is important in order to ensure that Basal Stimulation is implemented in in the long run?	Which obstacles do you perceive?<br>What could facilitate the long-term implementation?<br>What requires additional concern?

Conclusion

Would you like to add something that seems important to you – something we have not talked about in the interview?

Saying thank you for the interview.

<u>Procedure of the interview (case vignette and interview guideline): nursing experts</u>

Introduction

- Introducing oneself to the participant.
- Informing about the aim: We would like to explore how interventions in the field of dementia care are implemented and what kind of challenges are associated with this in practice.
  By the term «intervention» we mean a nursing activity or a concept that has been implemented in practice with the aim to improve the quality of care for people with dementia.
- To figure this out, we would like to interview nursing managers and persons who implement interventions in nursing homes.
- Informing about the procedure: First, I will ask you to read a story, to immerse yourself into this story and to tell me what you think about it. After that, I would like to ask some more detailed questions. You are welcome to bring in your point of view as nursing experts. I will record this interview in order to transcribe it afterwards.
- The interview will take approximately one and a half hours.
- Do you have any questions before we start?
- *(Procedure: We handed out the vignette and asked the participants to read it. Then we presented a graph about Basal Stimulation and briefly explained each of the points)*

Case vignette

Miss Schmid is responsible for initiating and implementing projects in a nursing home in Eastern Switzerland. At a congress of the Swiss Professional Associations of Nurses she listened to a lecture about Basal Stimulation. Since then, she is convinced that by means of this concept persons with dementia could experience better care. So far, in her institution no actions have been taken in this direction. This is a cause for concern and Miss Schmid is alarmed. She cannot get out of her mind the possibilities associated with this concept for the residents in her institution. She expects positive consequences, for example improved body awareness and reduced agitation. Probably her colleagues could also benefit from this concept, particularly with regard to interventions in the context of physical care for persons with aggressive or agitated behavior. The whole institution might have a benefit since Basal Stimulation could also have an impact on the attitude of all professionals. Miss Schmid deliberates whether it would be possible to integrate this concept on the specialized ward for persons with dementia.
What should she do? How could she realize this? On the next day during a coffee break, she meets her superior, the nursing manager and enthusiastically tells her about the lecture and her ideas …

Procedure: First, we ask the guiding questions and wait for the answers. Afterwards, we ask the concrete questions, as far as they are still necessary.

Guiding questions	Concrete questions
How would you launch the project?	What are the focal points of your deliberations?<br>Which persons on which levels would you involve?<br>Which steps seem important to you?<br>Which resources do you need?<br>What do you have to take into consideration (principally)?<br>What requires particular consideration?<br>Which basic conditions are required?<br>How did you proceed in similar projects?
Which obstacles or barriers do you perceive?	Which obstacles do you perceive<br>… on the organizational level?<br>… on the level of nurses/health professionals?<br>… on the level of persons with dementia?<br>… on the level of relatives/on the family level?
What could facilitate the implementation of the project from your point of view?	What facilitates the implementation of the project<br>… on the organizational level?<br>… on the level of nurses/health professionals?<br>… on the level of persons with dementia?<br>… on the level of relatives/on the family level?
What is particularly important to successfully implement the project from your point of view?	Which aspects are significant from your point of view<br>… on the organizational level?<br>… on the level of nurses/health professionals?<br>… on the level of persons with dementia?<br>… on the level of relatives/on the family level?
How do you recognize the successful implementation of the project?	How do you recognize the successful implementation of the project<br>… on the organizational level?<br>… on the level of nurses/health professionals?<br>… on the level of persons with dementia?<br>… on the level of relatives/on the family level?
How do you ensure that Basal Stimulation is implemented in the long run?	Which obstacles do you perceive?<br>What could facilitate the long-term implementation?<br>What requires additional concern?

Conclusion

Would you like to add something that seems important to you – something we have not talked about in the interview?

Saying thank you for the interview.
